# Verification of the Efficacy and Safety of Qi-Replenishing Chinese Medicine in Treating Prediabetes: A Meta-Analysis and Literature Review

**DOI:** 10.1155/2020/7676281

**Published:** 2020-11-10

**Authors:** Shujie Xia, Bizhen Gao, Shujiao Chen, Xuejuan Lin, Ping Zhang, Yujuan Chai, Candong Li, Tetsuya Asakawa

**Affiliations:** ^1^Research Base of Traditional Chinese Medicine Syndrome, Fujian University of Traditional Chinese Medicine, Fuzhou 350122, China; ^2^Department of Neurosurgery, Hamamatsu University School of Medicine, Handayama, Hamamatsu, Shizuoka, Japan; ^3^School of Medical Engineering, Health Science Center, Shenzhen University, Shenzhen 518060, China

## Abstract

**Background:**

Qi-replenishing Chinese medicines (QCMs) are used for treating prediabetes in the traditional Chinese medicine (TCM) clinical practice. The aims of this meta-analysis were to (i) verify the efficacy and safety of QCMs in treating prediabetes and (ii) investigate the clinical outcomes between the trials complying with and not complying with the principle of “syndrome differentiation.”

**Methods:**

We included only randomized controlled clinical trials (RCTs) whose Jadad scores were not less than 4. The overall clinical outcomes, including the incidence rate of diabetes, normalization of blood glucose, changes in fasting blood glucose (FBG), 2 h postprandial blood glucose, HbA1c, and occurrence of adverse events, were evaluated. Subgroup analyses were performed.

**Results:**

A total of 11 RCTs that enrolled 2210 patients with prediabetes were included. We observed that overall treatment with QCMs significantly ameliorated the clinical outcomes of prediabetes without increasing incidence of adverse events. The results of subgroup analyses revealed that prescribing QCMs complying with syndrome differentiation ameliorated all the clinical indices, whereas prescribing not complying with syndrome differentiation could not achieve significant amelioration in FBG and HbA1c levels. Furthermore, the subgroup with syndrome differentiation reported more adverse events.

**Conclusions:**

The overall results suggested that QCMs are effective and safe in treating prediabetes. Results of subgroup analyses indicated that the groups with syndrome differentiation presented better efficacy but had a higher occurrence of adverse events. This study indicated the important role of the principle of syndrome differentiation in TCM and that the adverse events of QCMs cannot be ignored in TCM clinical practice.

## 1. Introduction

Diabetes mellitus (DM) is a global public health concern with a high prevalence rate. Prediabetes is characterized by mild impaired fasting blood glucose (IFG) and/or impaired glucose tolerance (IGT). Although prediabetes generally does not cause any noticeable symptoms, it can easily progress to type 2 diabetes (T2D) if it remains unaware of or is neglected. Saklayen reported that approximately one-fourth of patients with T2D neglected that they have prediabetes [1], which may leave T2D in an “untreated” state. This untreated T2D is extremely dangerous, as it is closely associated with the onset of heart disease or stroke. Our previous study also elucidated a dangerous pathophysiologic manifestation in those untreated patients with metabolic syndrome [2]. Hence, it is crucial to intervene in this nonsymptomatic prediabetes. Studies have documented that early interventions in prediabetes can prevent or delay the progression of T2D and the development of complications [3, 4]. In this regard, conventional interventions include lifestyle modification (LM) or/and antidiabetic medicine. However, such interventions are far from satisfactory. This is because antidiabetic medicines, such as metformin and acarbose, may cause adverse events, and their effects are short-lived. LM must be insisted on for a long-term period, which cannot be achieved by some people. Therefore, some alternative therapies are being considered. In China, herbs based on the theories of traditional Chinese medicine (TCM) are commonly used for treating patients with prediabetes to prevent its progression to T2D. It has been well documented that such herbs contribute to reduce the incidence of T2D, lower the blood glucose (BG) levels, and ameliorate obesity [5, 6]. A special type of TCM herbs, namely, Qi-replenishing Chinese medicines (QCMs), are widely used for treating prediabetes [7, 8]. Several studies have also claimed better efficacy of QCMs in treating prediabetes by enhancing insulin sensitivity [9], reducing inflammatory response [10], and correcting glucose and lipid metabolism disorders [11, 12].

On the other hand, “holistic concept” and “syndrome differentiation” are two basic principles in TCM [13]. “Syndrome differentiation” requires that the herbs and other therapies be selected according to the TCM syndrome. In China, most of the clinicians practicing TCM comply with “syndrome differentiation” in their clinical practice. However, in other countries such as Japan, the TCM herbs are generally selected according to the disease or symptoms, rather than the differentiation of the TCM syndrome. Therefore, whether the clinicians comply with the principle of “syndrome differentiation” remains controversial.

Herein, we designed and conducted a meta-analysis and systematic literature review strictly following the guidelines of the Preferred Reporting Items for Systematic Reviews and Meta-Analyses (PRISMA) [14]. Our aims in this study were concerned with two aspects as follows. (i) The first aspect is verifying the efficacy and safety of QCMs in treating prediabetes; for this purpose, only randomized controlled clinical trials (RCTs) with a rigorous experimental design were included in this study. We attempted to obtain convincing evidence regarding QCMs. (ii) The second aspect is comparing the clinical outcomes between the trials complying with and not complying with the principle of “syndrome differentiation.” Our purpose was to determine whether “syndrome differentiation” is actually indispensable in TCM clinical practice. We believe that our study findings may contribute to further understanding of the value of QCMs in treating prediabetes as well as the importance of the principle of “syndrome differentiation” in TCM.

## 2. Methods

### 2.1. Literature Search Strategy

A comprehensive electronic search was performed in five English databases (the Web of Science, Medline, Cochrane Library, PubMed, and Embase) and three Chinese databases (Chinese National Knowledge Infrastructure, VIP Information Database, and Wanfang Database) from inception to January 2020. The medical subject heading (MeSH) terms “Prediabetic State” and “Drugs, Chinese Herbal,” and the publication type of “Randomized controlled trial” were used. We used terms including [“prediabetic States” OR “prediabetes” OR “impaired fasting glucose” OR “impaired glucose tolerance”] AND [“traditional Chinese medicine” OR “Chinese medicinal herbs” OR “Chinese herbal medicine” OR “decoction” OR “Chinese patent medicine” OR “Chinese patent drug” OR “Replenishing Qi Chinese herbs”] AND [“randomized” OR “placebo”]. References of important articles were manually searched for acquiring possible relevant studies. The websites of the international clinical trial registry (available at http://clinicaltrials.gov/) and the Chinese clinical trial registry (available at http://www.chictr.org.cn/index.aspx) were also explored to find unpublished studies.

Studies were included and excluded based on the PICOs criteria, which are presented in Table 1. Only studies with high quality (Jadad scores ≥4) [15] were included in analysis.

### 2.2. Data Extraction and Evaluation

First, two authors (SX and BG) independently screened the identified records by reading the title and abstract. The remaining articles were subsequently evaluated by a third author (SC) by reading the full text, and then the study quality was evaluated using the Jadad score [15]. Finally, the quality of the included articles was cross-checked and confirmed by a senior researcher (TA). Next, data from eligible studies were extracted and recorded independently by two authors (XL and CL). The following data were extracted from each study: primary author, title, year of publication, study design, study population, number of patients, duration of study, TCM intervention method, baseline and endpoint values of outcome measure, and details of treatment and control. All the data were finally assessed by a third-party author (YC) before subjecting them to meta-analysis.

Two authors (SC and CL) independently evaluated the risk of bias according to the Cochrane Handbook for Systematic Reviews of Interventions. The following items were considered: random sequence generation, allocation concealment, blinding of participants and personnel, blinding of outcome assessment, incomplete outcome data, selective reporting, and other bias. The bias in each domain was judged as low risk, high risk, and unclear risk of bias. Discussions were performed on a weekly basis to resolve disagreements and finally reach the consensus.

### 2.3. Statistical Analysis

The present study was strictly conducted following the guidelines provided by Cochrane Handbook [16] and the PRISMA guidelines [14]. The RevMan 5.3 software was used for the meta-analysis. Continuous data were pooled to estimate the weighted mean differences (WMDs) and were accompanied by 95% confidence intervals (CIs). Categorical data were pooled to determine the relative risks (RRs) and were accompanied by 95% CIs. The *I*^*2*^ statistics were used to measure heterogeneity. Regarding the homogeneity test, when *p* > 0.1 and *I*^*2*^ ≤50%, the trials were considered to be homogeneous, and a fixed-effects model was used. However, when *p* < 0.1 or *I*^*2*^ >50%, the trials were regarded as heterogeneous and then a random-effects model was applied. To gain better understanding of the efficacy of QCMs, we performed a subgroup analysis. Subgroups were established according to (i) the experimental design involved in the included studies, namely, QCM + lifestyle modification (LM) versus LM or QCM + LM versus placebo/metformin + LM, and (ii) methods of prescribing the herbs, namely, prescription complying with the principle of syndrome differentiation or prescription without complying with the principle of syndrome differentiation. Sensitivity analysis was conducted by mutual conversion between a random-effects model and a fixed-effects model to evaluate the stability of the research. In addition, the studies were assessed in sequence to further identify possible sources of heterogeneity.

## 3. Results

### 3.1. Results of Literature Search

**Figure 1:**
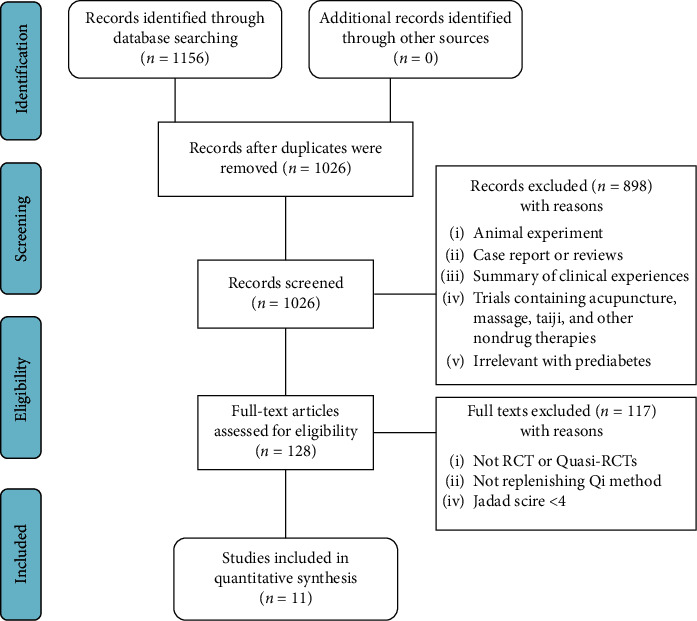
Flow chart of the searching strategy and the literature selection.

A total of 1156 articles were obtained. First, 130 articles were removed due to repetition. Second, 898 articles were excluded due to the following reasons: (i) animal experiments; (ii) case reports or reviews; (iii) trials containing acupuncture, massage, Taiji, and other nondrug therapies; and (iv) not pertaining to prediabetes. The remaining 128 articles were submitted for full-text assessment, in which 117 were removed due to the following reasons: (i) no RCTs or quasi-RCTs; (ii) not involving the Qi-replenishing method; and (iii) Jadad score <4. Finally, a total of 11 eligible studies were included in the present meta-analysis [17–27] (Figure 1).

Table 2 shows the characteristics of the included studies. Eight RCTs were published in English [17, 18, 20–23, 25, 26], and the remaining three were in Chinese [19, 24, 27]. One study [17] was conducted in Australia, and the others were conducted in China. The sample size of the included studies ranged from 65 to 514, with 1116 patients in the treatment groups versus 1094 patients in the control groups. All the 11 studies reported baseline comparability. The duration of treatment ranged from 3 to 12 months. The follow-up duration in three studies [17, 22, 23] ranged from 2 to 24 months. One study did not report the occurrence of adverse events [27]. In five studies, TCM herbs were prescribed according to the conventional principle of syndrome differentiation [19, 21, 24–26], whereas this was not followed in the remaining six studies.

### 3.2. Assessment of Study Quality

**Figure 2:**
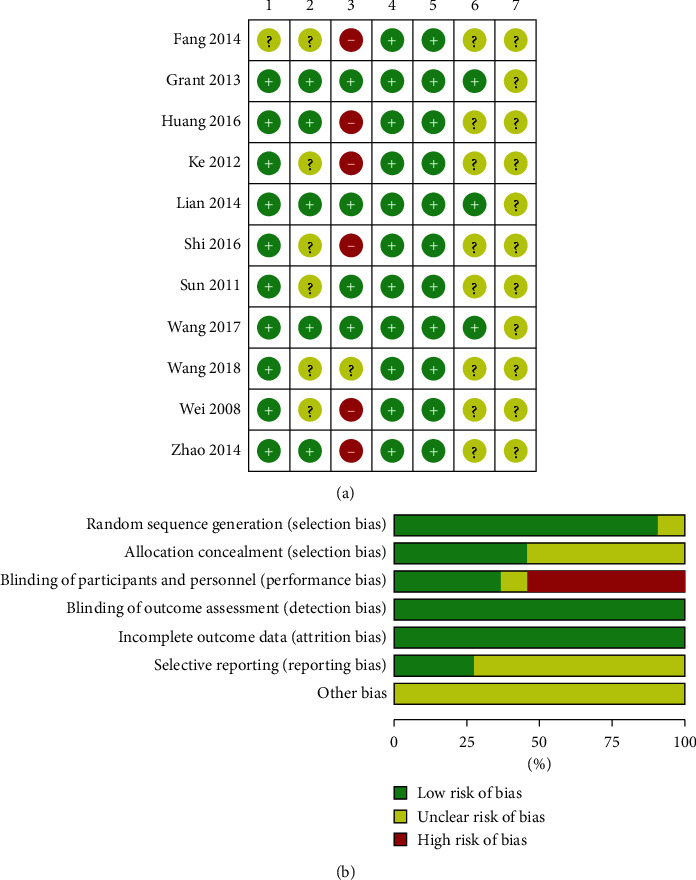
Potential bias risk involved in this study. (a) Risk of bias in the included studies 1 = random sequence generation (selection bias), 2 = allocation concealment (selection bias), 3 = blinding of participants and personnel (performance bias), 4 = blinding of outcome assessment (detection bias), 5 = incomplete outcome data (attrition bias), 6 = selective reporting (reporting bias), 7 = other bias. (b) Summary of the bias risk for the included studies.

We selected only studies with Jadad scores >4. The Jadad scores are listed in Table 2. The risk of bias is illustrated in Figure 2. Randomization was reported in all 11 studies, with 6 studies reporting the method of random sequence generation using computer software [17, 18, 20, 22–24] and 4 studies reporting it using random number tables [19, 21, 25, 27]. Only one study did not describe the randomization method in detail [26]. Five trials reported the method of allocation concealment [17, 18, 22–24], and four trials reported the blinding of participants and personnel [17, 18, 22, 27]. All studies reported about drop-outs or withdrawals. Detection bias in these trials was considered to be at low risk based on the objective outcome indexes. In three studies, the selective reporting bias was judged to be at low risk because their trial protocols were available [17, 18, 22]. As it was not possible to obtain relevant information concerning “sample calculation” and “conflicts of interest,” the other bias was judged to be “unclear” (Figure 2). As the number of included trials in each analysis was less than 10, the publication bias could not be evaluated [28]. With respect to sensitivity analyses, we found that *I*^*2*^ did not change in the mutual conversion, suggesting that the findings were stable. Based on the results of the subgroup analysis, various treatments used in control groups were considered as the primary source of heterogeneity. Furthermore, different QCM prescriptions, dosages, and follow-up duration may also potentially cause heterogeneity. Therefore, we screened all the articles and excluded items based on changes in heterogeneity. In the sensitivity analysis of FBG, 2hPG, and HbA1C levels, we found that three trials not complying with syndrome differentiation [17, 20, 23] were the primary sources of high heterogeneity, which may be associated with different selection of TCM herbs and small sample size.

### 3.3. Clinical Outcomes

#### 3.3.1. Efficacy of Preventing Prediabetes from Progressing to Diabetes

**Figure 3:**
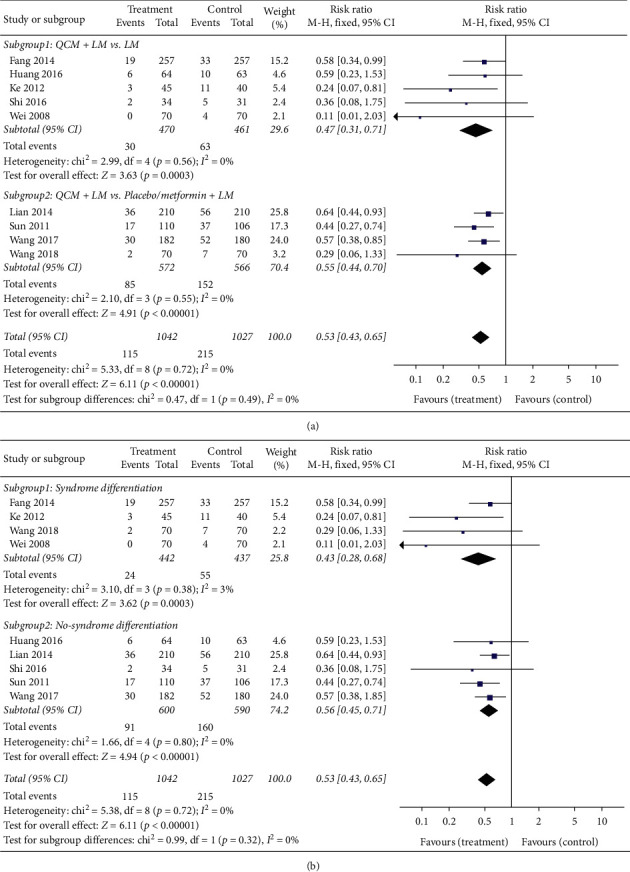
Meta-analysis for the efficacy of QCMs in reducing the incidence rate of diabetes. (a) Forest plot for comparisons between the subgroups QCM + LM vs. LM. QCM + LM and QCM + LM vs. placebo/metformin + LM (treatment group vs. control group). (b) Forest plot for comparisons in the subgroups complying with syndrome differentiation and not complying with syndrome differentiation (treatment group vs. control group). LM: lifestyle modification; QCM: Qi-replenishing Chinese Medicine.

Figure 3 illustrates the incidence rate during the follow-up period. The results of nine trials with 2069 cases indicated that the QCM groups exhibited a lower incidence rate of diabetes than the control groups, which suggested that QCM is helpful in preventing prediabetes from progressing to diabetes (*n* = 2069; RR = 0.53; 95% CI [0.43, 0.65]; *p* < 0.00001; *I*^*2*^ = 0%). As these trials exhibited nonsignificant heterogeneity, a fixed-effects model was used for statistical analysis. Regarding the subgroup analysis, five trials that compared QCM + LM versus LM reported a lower incidence rate (*n* = 931; RR = 0.47; 95% CI [0.31, 0.71]; *p* = 0.0003; *I*^*2*^ = 0%). The remaining four trials that compared QCM + LM versus placebo/metformin + LM also reported the same results (*n* = 1138; RR = 0.55; 95% CI [0.44, 0.70]; *p* < 0.00001; *I*^*2*^ = 0%) (Figure 3(a)). Four trials complying with syndrome differentiation reported that QCM groups had a lower incidence rate than control groups (*n* = 879; RR = 0.43; 95% CI [0.28, 0.68]; *p* = 0.0003; *I*^*2*^ = 3%). The remaining five trials not complying with syndrome differentiation also reported similar results; that is, QCM groups had a lower incidence rate than control groups (*n* = 1190; RR = 0.56; 95% CI [0.45, 0.71]; *p* < 0.00001; *I*^*2*^ = 0%) (Figure 3(b)).

#### 3.3.2. Normalization of Blood Glucose

**Figure 4:**
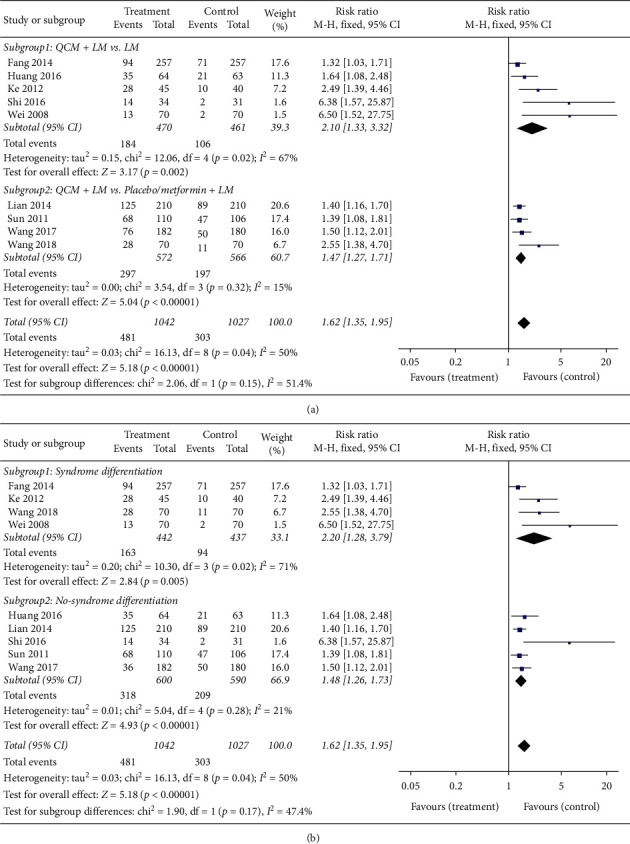
Meta-analysis for the efficacy of QCMs in normalizing the blood glucose level. (a) Forest plot for comparisons between the subgroups QCM + LM vs. LM. QCM + LM and QCM + LM vs. placebo/metformin + LM (treatment group vs. control group). (b) Forest plot for comparisons in the subgroups complying with syndrome differentiation and not complying with syndrome differentiation (treatment group vs. control group). LM: lifestyle modification; QCM: Qi-replenishing Chinese Medicine.

Figure 4 depicts the data indicating whether QCM was helpful in normalizing BG levels. In this regard, nine trials with 2069 cases were analyzed. The results showed that QCM contributed toward the normalization of BG levels (*n* = 2069; RR = 1.62; 95% CI [1.35, 1.95]; *p* < 0.00001; *I*^*2*^ = 50%). Due to the presence of heterogeneity among the trials, the results were analyzed by a random-effects model. In the subgroup analysis, five trials comparing QCM + LM versus LM showed that QCMs contributed toward normalizing the BG level (*n* = 931, RR = 2.10, 95% CI [1.33, 3.32], *p* = 0.002, *I*^*2*^ = 67%). The remaining four trials comparing QCM + LM versus placebo/metformin + LM reported similar results (*n* = 1138, RR = 1.47, 95% CI [1.27, 1.71], *p* < 0.00001, *I*^*2*^ = 15%) (Figure 4(a)). Four trials complying with syndrome differentiation demonstrated that QCM had better efficacy to normalize the BG level (*n* = 879, RR = 2.20, 95% CI [1.28, 3.79], *p* = 0.005, *I*^*2*^ = 71%). Five trials not complying with syndrome differentiation achieved the same results (*n* = 1190, RR = 1.48, 95% CI [1.26, 1.73], *p* < 0.00001, *I*^*2*^ = 21%) (Figure 4(b)).

#### 3.3.3. Reduction of Fasting Blood Glucose Levels

**Figure 5:**
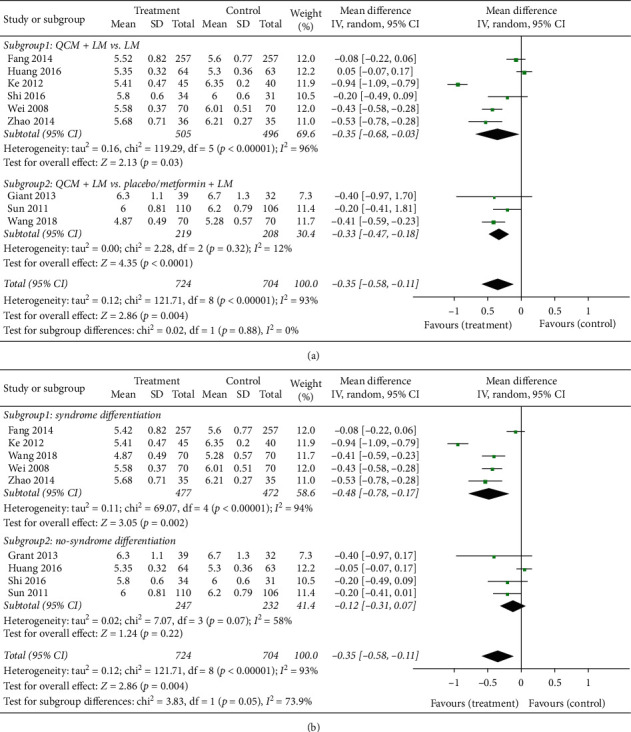
Meta-analysis for the efficacy of QCMs in reducing the fasting blood glucose level. (a) Forest plot for comparisons between the subgroups QCM + LM vs. LM. QCM + LM and QCM + LM vs. placebo/metformin + LM (treatment group vs. control group). (b) Forest plot for comparisons in the subgroups complying with syndrome differentiation and not complying with syndrome differentiation (treatment group vs. control group). LM: lifestyle modification; QCM: Qi-replenishing Chinese Medicine.

The results of reduction of FBG levels are shown in Figure 5. Nine studies with 1428 cases reported on FBG levels. Overall, QCM groups achieved better reduction of FBG levels (*n* = 1428; MD = −0.35; 95% CI [−0.58, −0.11]; *p* = 0.004; *I*^*2*^ = 93%). There was obvious heterogeneity between the trials, and hence a random-effects model was used for statistical analysis. With respect to the subgroup analysis, the results from six trials comparing QCM + LM versus LM indicated that QCM treatment groups achieved better reduction of FBG levels (*n* = 1001; MD = −0.35; 95% CI [−0.68, −0.03]; *p* = 0.03; *I*^*2*^ = 96%). The remaining three trials comparing QCM + LM versus placebo/metformin + LM reported the same results (*n* = 427; MD = −0.33; 95% CI [−0.47, −0.18]; *p* < 0.00001; *I*^*2*^ = 12%) (Figure 5(a)). Four trials complying with syndrome differentiation showed that QCM groups achieved better reduction of FBG levels (*n* = 949; MD = −0.48, 95% CI [−0.78, −0.17]; *p* = 0.002; *I*^*2*^ = 94%). Interestingly, the remaining five trials not complying with syndrome differentiation did not show any significant difference in the reduction of FBG levels between QCM treatment groups and control groups (*n* = 479; MD = −0.12; 95% CI [−0.31, 0.07]; *p* = 0.22; *I*^*2*^ = 58%) (Figure 5(b)).

#### 3.3.4. Changes in the 2 h Postprandial Blood Glucose Level

**Figure 6:**
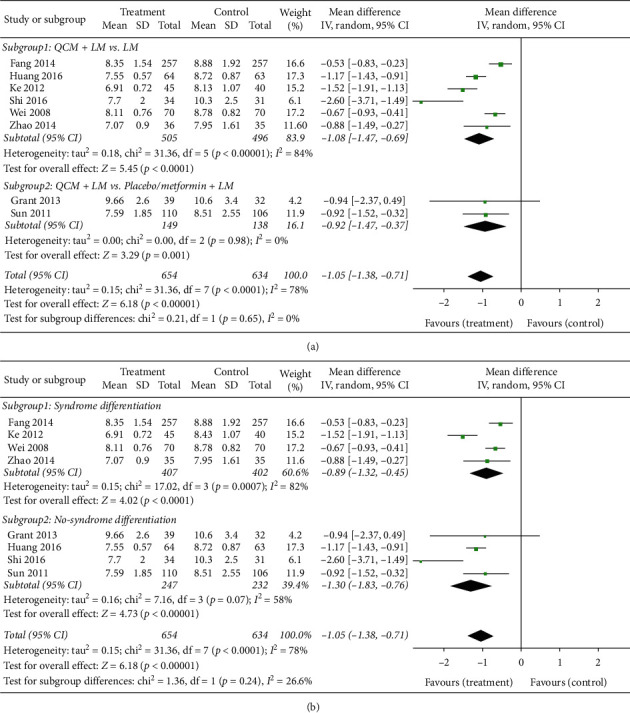
Meta-analysis for the efficacy of QCMs in reducing the 2 h postprandial blood glucose level. (a) Forest plot for comparisons between the subgroups QCM + LM vs. LM. QCM + LM and QCM + LM vs. placebo/metformin + LM (treatment group vs. control group). (b) Forest plot for comparisons in the subgroups complying with syndrome differentiation and not complying with syndrome differentiation. LM: lifestyle modification; QCM: Qi-replenishing Chinese Medicine (treatment group vs. control group).

Figure 6 depicts the results of changes in 2hPG levels. Eight studies with 1288 cases reported the changes in 2hPG levels. Overall, the QCM groups achieved better reduction of 2hPG levels (*n* = 1288; MD = −1.05; 95% CI [−1.38, −0.71]; *p* < 0.00001; *I*^*2*^ = 78%). As there was remarkable heterogeneity between the trials, a random-effects model was used for statistical analysis. Regarding the subgroup analysis, the results from six trials comparing QCM + LM versus LM indicated that QCM treatment groups achieved better reduction of 2hPG levels (*n* = 1001; MD = −1.08; 95% CI [−1.47, −0.69]; *p* < 0.00001; *I*^*2*^ = 84%). The remaining two trials comparing QCM + LM versus placebo/metformin + LM also achieved similar results (*n* = 287; MD = −0.92; 95% CI [−1.47, −0.37]; *p* = 0.001; *I*^*2*^ = 0%) (Figure 6(a)). Four trials complying with syndrome differentiation reported that QCM groups also achieved better reduction of 2hPG levels (*n* = 809; MD = −0.89, 95% CI [−1.32, −0.45]; *p* < 0.0001; *I*^*2*^ = 82%). The remaining four trials not complying with syndrome differentiation also reported similar results (*n* = 479, MD = −1.30, 95% CI [−0.83, −076], *p* < 0.0001, *I*^*2*^ = 58%) (Figure 6(b)).

#### 3.3.5. Changes in HbA1c Levels

**Figure 7:**
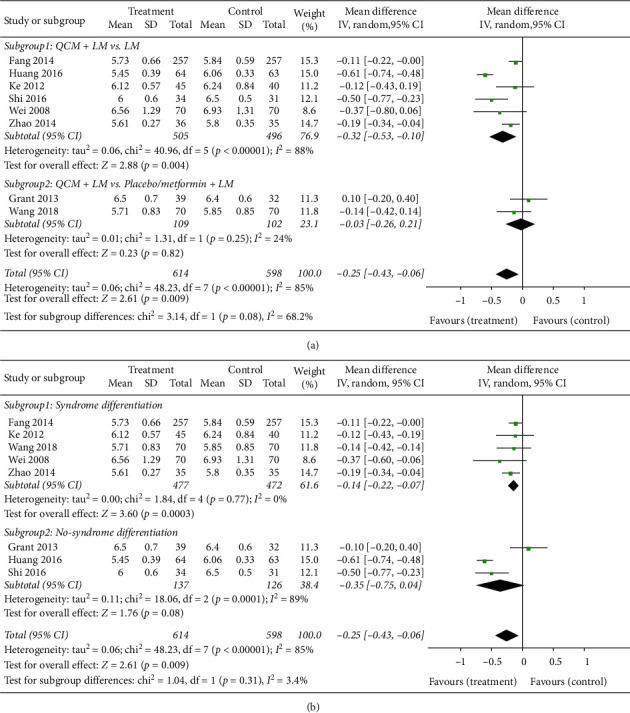
Meta-analysis for the efficacy of QCMs in reducing the HbA1c level. (a) Forest plot for comparisons between the subgroups QCM + LM vs. LM. QCM + LM and QCM + LM vs. placebo/metformin + LM (treatment group vs. control group). (b) Forest plot for comparisons in the subgroups complying with syndrome differentiation and not complying with syndrome differentiation. LM: lifestyle modification; QCM: Qi-replenishing Chinese Medicine (treatment group vs. control group).

The changes in HbA1c levels are shown in Figure 7. Eight studies with 1210 cases reported the changes in HbA1c levels. Overall, the QCM groups achieved better reduction of HbA1c levels (*n* = 1212; MD = −0.25; 95% CI [−0.43, −0.06]; *p* = 0.009; *I*^*2*^ = 85%). Due to the significant heterogeneity between trials, a random-effects model was used for statistical analysis. Regarding the subgroup analysis, the results from six trials comparing QCM + LM versus LM indicated that QCM treatment groups achieved better reduction of HbA1c levels (*n* = 1001; MD = −0.32; 95% CI [−0.53, −0.10]; *p* = 0.004; *I*^*2*^ = 88%). The remaining two trials comparing QCM + LM versus placebo/metformin + LM did not find any significant difference (*n* = 211; MD = −0.03; 95% CI [−0.26, −0.21]; *p* = 0.82; *I*^*2*^ = 24%) (Figure 7(a)). Four trials complying with syndrome differentiation reported that QCM groups achieved better reduction of HbA1c levels (*n* = 949; MD = −0.14, 95% CI [−0.22, −0.07]; *p* = 0.0003, *I*^*2*^ = 0%). Importantly, the remaining three trials not complying with syndrome differentiation did not report any significant difference in the reduction of HbA1c levels between QCM treatment groups and control groups (*n* = 263; MD = −0.35; 95% CI [−0.75, −0.04]; *p* = 0.08; *I*^*2*^ = 89%) (Figure 7(b)).

### 3.4. Adverse Events

**Figure 8:**
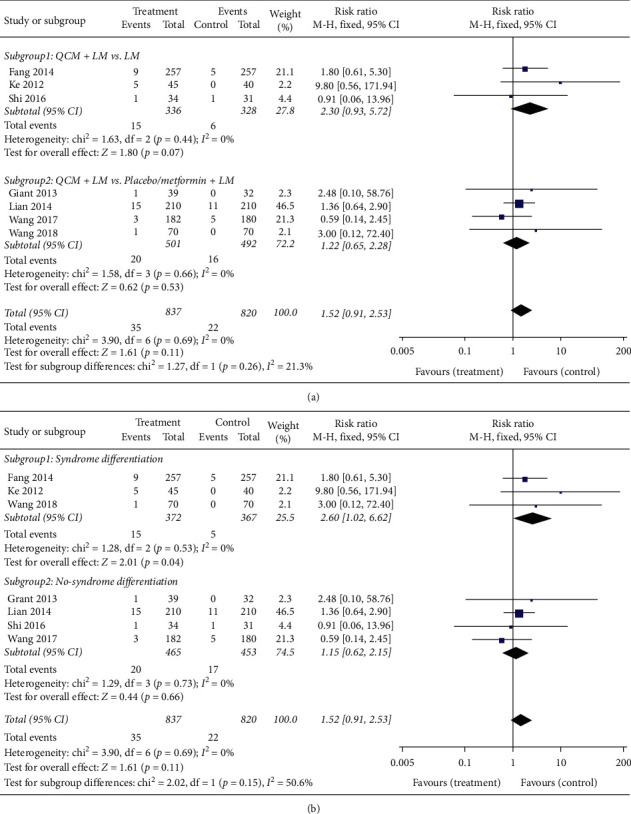
Meta-analysis for the occurrence of adverse events. (a) Forest plot for comparisons between the subgroups QCM + LM vs. LM. QCM + LM and QCM + LM vs. placebo/metformin + LM (treatment group vs. control group). (b) Forest plot for comparisons in the subgroups complying with syndrome differentiation and not complying with syndrome differentiation. LM: lifestyle modification; QCM: Qi-replenishing Chinese Medicine (treatment group vs. control group).

Ten trials reported on adverse events, among which three trials reported no occurrence of adverse events, and the remaining seven reported the occurrence of adverse events. The most common adverse events were gastrointestinal reactions, dizziness, and weakness. Overall, there was no significant difference between QCM groups and control groups in the occurrence of adverse events (*n* = 1657; MD = 1.52; 95% CI [0.91, 2.53]; *p* = 0.11; *I*^*2*^ = 0%). In the subgroup analysis, the results from three trials comparing QCM + LM versus LM indicated that no significant difference was found between QCM groups and control groups (*n* = 664; RR = 2.30; 95% CI [0.93, 5.72]; *p* = 0.07; *I*^*2*^ = 0%). The remaining four trials comparing QCM + LM versus placebo/metformin + LM also reported similar results (*n* = 991; RR = 1.22; 95% CI [0.65, 2.28]; *p* = 0.53; *I*^*2*^ = 0%) (Figure 8(a)). Three trials complying with syndrome differentiation demonstrated that there was a significant difference in the occurrence of adverse events between QCM groups and control groups (*n* = 739; RR = 2.60; 95% CI [1.02, 6.62]; *p* = 0.04; *I*^*2*^ = 0%). The remaining four trials not complying with syndrome differentiation indicated that no difference existed in the occurrence of adverse events between QCM groups and control groups (*n* = 918; RR = 1.15; 95% CI [0.62, 2.15]; *p* = 0.66; *I*^*2*^ = 0%) (Figure 8(b)).

## 4. Discussion

In the present investigation, we conducted a meta-analysis to verify the efficacy and safety of QCMs in treating prediabetes. Overall, we observed that treatment with QCMs significantly ameliorated the clinical outcomes of prediabetes. Meanwhile, we did not find any significant difference in the occurrence of adverse events between QCM groups and control groups in the overall data. Thus, the efficacy and safety of QCMs in treating prediabetes were verified. Importantly, in the subgroup analysis, we found that prescribing QCMs complying with syndrome differentiation resulted in significant amelioration of all the indices of prediabetes. However, prescribing QCMs not complying with syndrome differentiation could not achieve significant amelioration in FBG and HbA1c levels. Therefore, we believe that complying with the principle of syndrome differentiation can lead to better efficacy in treating prediabetes. Furthermore, the subgroup with syndrome differentiation had a higher occurrence of adverse events, which indicated that the adverse events of traditional medicines cannot be ignored. To the best of our knowledge, this is the first meta-analysis to elucidate the importance of the principle of syndrome differentiation. We believe that the findings of the present study can provide convincing evidence regarding the efficacy and safety of QCMs in treating prediabetes and the crucial role of syndrome differentiation in TCM practice.

### 4.1. Evidence of Efficacy and Safety of QCMs

We verified various indices related to the clinical outcomes of prediabetes. First, we found that QCMs led to a significant lower incidence rate of T2D during the follow-up period (2–24 months) (Figure 3). Both the overall data and the subgroup analysis provided the same results, thereby suggesting that QCMs can prevent the progression of prediabetes to T2D. In addition, QCMs significantly normalized the BG levels (Figure 4) and reduced the overall levels of FBG (Figure 5), 2hPG (Figure 6), and HbA1c (Figure 7). Therefore, QCMs improved the worsened indices of prediabetes in comparison with patients undergoing LM and/or placebo/metformin. These ameliorations led to a comprehensive result, that is, the reduction in the incidence rate of T2D. The evidence obtained in this investigation confirmed that QCMs are a better selection in addition to the conventional LM and antidiabetic medicine for treating subjects with prediabetes.

Furthermore, the overall analysis of adverse events did not indicate that QCMs caused more adverse events than the conventional LM and/or placebo/metformin (Figure 8), thereby confirming the safety of QCMs. Still, importantly, in the subgroup analysis, we did find that the subgroup with syndrome differentiation exhibited higher occurrence of adverse events than the groups not using QCMs (Figure 8(b)). These results indicated that the adverse events of QCMs must be seriously considered in clinical practice. Currently, there is an increasing focus on the adverse events of traditional medicines. Although several clinicians practicing TCM traditionally believe that TCM treatments have no or few adverse effects [13, 29], several reports have emphasized that adverse events do occur in TCM treatments. For instance, Ng et al. reported on the adverse events of aristolochic acids in several herbal medicines [30]. One of our previous studies also discussed the potential adverse events such as the occurrence of melanosis coli when using herbal medicines for treating cholelithiasis [29]. Results of the present study have reconfirmed the occurrence of adverse events in the subgroup with syndrome differentiation. In fact, the safety of traditional medicines also requires rigorous verification, which is similar to the use of Western medicine. As our previous study indicated that “lack of reporting of adverse events and follow-up” has been a primary nonsystematic problem encountered in several TCM studies [13], based on the results of the present study, we suggest that the adverse events of TCM cannot be ignored.

### 4.2. Principle of Syndrome Differentiation: Is It Really Important for TCM?

Another important finding was derived from our subgroup analysis. Although the results of the incidence rate, normalization of BG levels, and changes in 2hPG levels indicated that both trials with and without complying with syndrome differentiation achieved significant improvements, we found that the subgroup with syndrome differentiation achieved significant improvement in terms of FBG (Figure 5(b)) and HbA1c (Figure 7(b)) levels, whereas the subgroup without syndrome differentiation did not. FBG and HbA1c are very important indices reflecting the fluctuation of basal and long-term blood glucose levels in both T2D and prediabetes. Although we did not directly compare the data between the subgroups with and without syndrome differentiation, our results provided indirect evidence indicating that patients using TCM prescription on the basis of syndrome differentiation achieved better efficacy. It is well known that the principle of syndrome differentiation is one of the most important characteristics of TCM. In other countries, such as Japan, TCM herbs are always selected based on the symptom or disease. Even in China, this scenario can be seen when TCM herbs are prescribed by a clinician practicing Western Medicine. Our results also indicated that prescribing without complying with the principle of syndrome differentiation can also achieve efficacy; however, in case these TCMs were selected by complying with the principle of syndrome differentiation, the efficacy might be better. We have provided indirect evidence regarding the importance of the principle of syndrome differentiation in the clinical practice of TCM. We intend to design a study directly comparing the efficacy and safety of trials with and without complying with the principle of syndrome differentiation in our future investigation.

### 4.3. Strength of the Evidence

The quality of studies of alternative therapy, including TCM, is always questioned due to the flaws in the experimental design [13, 29, 31]. To obtain convincing evidence, we selected only those trials whose Jadad scores were ≥4. Randomization, control, and blinding (or using objective indices [13]) were designed in the involved studies. Furthermore, in some trials, the experiment was designed using “T + C vs. C + P (T: therapy of traditional medicine; C: conventional treatments; P: placebo), which has been described in our previous studies [13, 29, 31]. Of the included 11 studies, 10 reported on adverse events. Five of the 11 studies reported allocation concealment. Systematic problems and nonsystematic problems that were defined in our previous study [13] were not found in the articles included in this meta-analysis. Hence, the quality of these included studies was satisfactory, and the meta-analysis conducted on the basis of these trials is convincing.

### 4.4. Limitations of the Evidence

Although most of the problems such as the flaws in the experimental design, which were well documented in our previous studies [13, 29, 31, 32], have been improved in the studies included in the present meta-analysis, the evidence obtained from this study suffers from the following limitations. (i) We included only 11 trials, and the sample size in some trials was small (Table 2). (ii) The follow-up period (2–24 months) was extremely short to draw a rigorous conclusion, because the progression from prediabetes to T2D is generally chronic. (iii) As described in our previous study, complete blinding and allocation concealment are very difficult to achieve in studies on alternative therapy. Using an objective index during the clinical observation might be a better solution [13]. With further advancements in TCM and education of evidence-based medicine, we believe that the quality of studies in TCM could improve. Therefore, we expect more well-designed, multicenter RCTs with large sample sizes, long-term follow-up, and objective indices in the future, which can provide more reliable evidence.

## 5. Conclusions

We designed and conducted a meta-analysis to verify the efficacy and safety of QCMs in treating prediabetes. Our overall results showed that QCMs significantly improved the incidence rate and normalized the levels of BG, FBG, 2hPG, and HbA1c in patients with prediabetes, but they did not improve the occurrence of adverse events. The efficacy and safety of QCMs were verified. In addition, we found that trials complying with syndrome differentiation achieved significant improvement in all the indices, whereas trials not complying with syndrome differentiation could not significantly improve FBG and HbA1c levels. These findings provided an indirect evidence demonstrating the importance of the principle of syndrome differentiation in the clinical practice of TCM. Furthermore, the subgroup with syndrome differentiation exhibited a higher occurrence of adverse events than patients not using QCMs, which suggested that the adverse events of QCMs cannot be ignored. These findings are helpful in understanding the clinical value of QCMs in treating prediabetes, along with the significance of complying with the principle of syndrome differentiation in TCM. Moreover, the adverse events of TCM cannot be ignored.

## Figures and Tables

**Table 1:** PICOS criteria for inclusion and exclusion of literatures in this study.

Parameters	Inclusion criteria	Exclusion criteria
Patients	Patients with prediabetes	Patients with diabetes, or patients without prediabetes.
Intervention	Treatment using qi-replenishing Chinese medicine	Treatment without using qi-replenishing Chinese medicine
Comparison	Treatment using lifestyle modification (LM), or LM + metformin, or LM + placebo	Treatment without using LM
Outcome	Incidence of diabetes, or normalization of blood glucose (NBG), or fasting blood glucose (FBG), or 2 h postprandial blood glucose (2hPG), or HbA1c, or adverse event	Without using the indices of incidence of diabetes, NBG, FBG, 2hPG, HbA1c and adverse event
Study design	Randomized controlled trials (RCTs) and Jadad score ≥4	Non-RCT or Jadad score <4

**Table 2:** Characteristics of included studies.

Studies	Jadad scores	Syndrome differentiation	Sample size		Age (year)		Intervention		Treatment period	Follow-up	Outcome measure	Adverse events	
			T	C	T	C	T	C				T	C
Fang 2014	4	Yes	257	257	54.95 ± 9.50	54.61 ± 10.51	Shenzhutiaopi granule	LM	12 m	NR	a, b, c, d, e, f	9	5
Grant 2013	7	No	39	32	58.3	59.9	Jiangtangxiaozhi capsules	Placebo + LM	4 m	2m	c, d, e, f	1	0
Huang 2016	5	No	64	63	52.02 ± 8.60	51.05 ± 9.25	Tangyiping granules	LM	3 m	24 m	a, b, c, d, e, f	0	0
Ke 2012	4	Yes	45	40	46.5 ± 7.3	45.7 ± 7.5	Lingguizhugan decoction	LM	6 m	NR	a, b, c, d, e, f	5	0
Lian 2014	7	No	210	210	52.95 ± 10.06	51.86 ± 10.16	Tianqi capsules	Placebo + LM	12 m	NR	a, b, f	15	11
Shi 2016	4	No	34	31	47.1 ± 7.1	49.9 ± 7.2	Jinlida granule	LM	3 m	NR	a, b, c, d, e, f	1	1
Sun 2011	4	No	110	106	51.0 ± 9.3	51.4 ± 9.5	Tianqi capsules	Placebo + LM	12 m	NR	a, b, c, d	NR	NR
Wang 2017	7	No	182	180	55.49 ± 8.61	53.49 ± 8.85	Jinqijiangtang tablet	placebo + LM	12 m	12 m	a, b, f	3	5
Wang 2018	4	Yes	70	70	43.7 ± 5.8	42.9 ± 6.1	Tangqianping granule	Metformin + LM	3 m	NR	a, b, c, e, f	1	0
Wei 2008	4	Yes	70	70	51.3 ± 8.8	50.7 ± 8.1	Tang no. 1 granule	LM	6 m	NR	a, b, c, d, e, f	0	0
Zhao 2014	5	Yes	35	35	46.49 ± 12.63	44.02 ± 12.93	Xiaotangsevenherb formula	LM	3 m	NR	c, d, e, f	0	0

LM: lifestyle modification; NR: not report; T: treatment group; C: control group; a: incidence of diabetes; b: normalization of blood glucose; c: fasting blood glucose (FBG); d: 2 h postprandial blood glucose (2hPG); e: hemoglobin A1c (HbA1c); f: adverse event.

## Data Availability

The data used to support the findings of this study are included within the article.
